# Mapping the expression of the sex determining factor *Doublesex1* in *Daphnia magna* using a knock-in reporter

**DOI:** 10.1038/s41598-017-13730-4

**Published:** 2017-11-02

**Authors:** Quang Dang Nong, Nur Syafiqah Mohamad Ishak, Tomoaki Matsuura, Yasuhiko Kato, Hajime Watanabe

**Affiliations:** 1 Department of Biotechnology, Graduate School of Engineering, Osaka University, Suita, Osaka, Japan; 2 Biotechnology Global Human Resource Development Program, Division of Advanced Science and Biotechnology, Department of Biotechnology, Graduate School of Engineering, Osaka University, Suita, Osaka, Japan; 3 Frontier Research Base of Global Young Researchers, Graduate School of Engineering, Osaka University, Suita, Japan

## Abstract

Sexually dimorphic traits are common and widespread among animals. The expression of the Doublesex-/Mab-3-domain (DM-domain) gene family has been widely studied in model organisms and has been proven to be essential for the development and maintenance of sex-specific traits. However, little is known about the detailed expression patterns in non-model organisms. In the present study, we demonstrated the spatiotemporal expression of the DM-domain gene, *doublesex1* (*dsx1*), in the crustacean *Daphnia magna*, which parthenogenetically produces males in response to environmental cues. We developed a *dsx1* reporter strain to track *dsx1* activity *in vivo* by inserting the *mCherry* gene into the *dsx1* locus using the TALEN-mediated knock-in approach. After confirming *dsx1* expression in male-specific traits in juveniles and adults, we performed time-lapse imaging of embryogenesis. Shortly after gastrulation stage, a presumptive primary organiser, named cumulus, first showed male-specific *dsx1* expression. This cell mass moved to the posterior growth zone that distributes *dsx1-*expressing progenitor cells across the body during axial elongation, before embryos start male-specific *dsx1* expression in sexually dimorphic structures. The present study demonstrated the sex-specific *dsx1* expression in cell populations involved in basal body formation.

## Introduction

Sexual reproduction is favoured by eukaryotes to produce offspring. At the gamete level, the sex can be either male or female^1,2^. However, substantial diversity has been recorded in sex-associated phenotypes and in the pathways of sex determination. The rapid evolution of a conserved biological process has raised a fundamental question on the appearance and advancement of sex and sex-limited traits through the history of the living world. In the search for the origin of sex, numerous studies have been conducted, which focus on elucidating the molecular basis of sexual development in various species^3^.

The discovery of the Doublesex-/Mab-3-domain (DM-domain) gene family has revealed a common node in the network of sex determination pathways^4,5^. Identified from a surprisingly wide range of taxa, members of the DM-domain gene family share a conserved DNA-binding domain known as the DM-domain. In mammals, DMRT1 primarily promotes and maintains the development of testes^6^. In *Drosophila*, Doublesex (DSX) controls the development of almost all sex-specific somatic aspects together with Fruitless (FRU)^7^. Although several divergent roles of DM-domain genes have been described in other species, most of these support sexual function in some way^5^. Thus, this gene family appears to have ancient origins and to have flexibly evolved through time. The spatial and temporal expressions of these genes have been exhaustively examined, focusing mainly on the fruit fly^8–11^, nematode^12^, and mouse^13,14^, indicating that their expressions are indicators of cells with the potential to show sex differences.

The cladoceran crustacean *Daphnia magna* presents three major advantages when used as a model species for the analysis of mechanisms underlying the development and evolution of sexually dimorphic traits. First, the cladoceran clade, arising from as early as the Devonian period^15^, is a possible common ancestor of Pancrustaceans, including insects and crustaceans^16^. This renders *Daphnia* a useful model for deducing the origin and evolution of a sex-determining pathway. Second, *Daphnia* uses a unique sex-determining system, where environmental cues are primarily considered for sex determination, stimulating germ cells at the late stage of oogenesis and leading to the development of males that are genetically identical to females^17^. Third, we recently developed methods for targeted gene disruption using Crispr/Cas9^18^ and TALEN systems in *D*. *magna* ^19^. We also succeeded in TALEN-mediated knock-in of DNA fragments and plasmid DNA *via* homologous recombination^20^ and non-homologous end joining (NHEJ)^21^, respectively. Along with available EST and genome sequences^22^, these genome editing tools would be useful for analysing orthologues of DM-domain genes in this species.

We previously identified five DM-domain genes within the *D*. *magna* genome^23,24^. Among these, an orthologue of *Drosophila dsx*, namely *dsx1*, is exclusively expressed in male-specific structures, and is involved in the control of male trait development^24^. Knock-down of *dsx1* in males feminised both somatic cells and germ cells. This suggested that the sexual fates of most cells in *Daphnia* were directly dependent on *dsx1* expression, as previously observed in *Drosophila*. However, this previous study shows limitations, as it only provides snapshot images of *dsx1* at certain time points^24^. In the present study, we proposed a real-time tracking tool and ultimately established a complete atlas of *dsx1* activity throughout *D*. *magna* development, focusing mainly on early embryogenesis. Using a genome editing technique, we successfully introduced an *in vivo* fluorescent reporter into the *dsx1* locus using the TALEN-mediated knock-in approach.

## Results

### Temporal expression of *dsx1* during development

**Figure 1:**
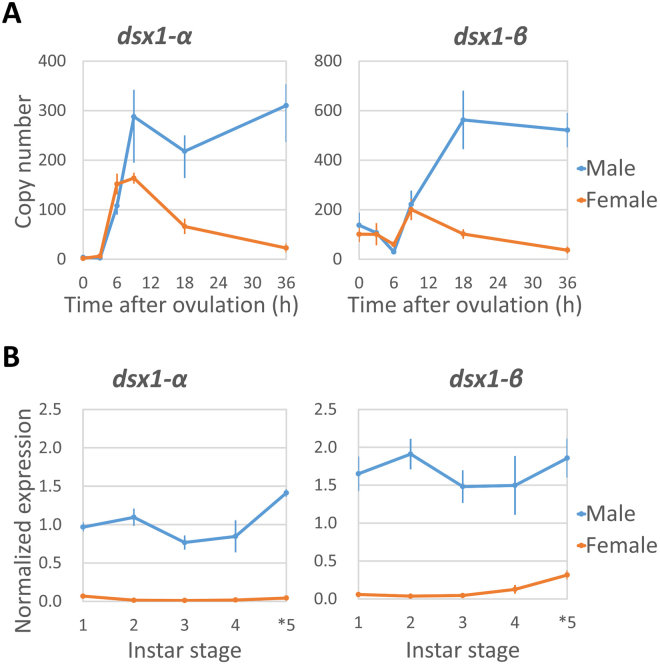
Expression of *dsx1* during development. (**A**) Expression during embryogenesis. Samples of males and females at 0, 3, 6, 9, 18, and 36 h post-ovulation were collected and the expression of *dsx1* transcripts was quantified. Primer pairs specific to the 5′ UTR of each transcript were used. N = 3. Error bars indicate standard error of the mean (SEM). (**B**) Expression during daphniid maturation. Samples of males and females of 72 (instar 1)-, 96 (instar 2)-, 120 (instar 3)-, 144 (instar 4)-, and 168 (instar 5 for male and instar 4 for female)-hour-old individuals were collected, and the expression levels of the two *dsx1* transcripts were quantified. Primer pairs specific to the 5′ UTR of each transcript were used. Normalisation was performed using *L32* (a ribosomal protein-encoding gene) expression as the internal control. N = 3. Error bars indicate SEM.

Sexually dimorphic expression of *dsx1* was described in a previous study^24^, in which the maternally inherited *dsx1-β* transcript was present at similar levels in male and female eggs. However, in that study^24^, both *dsx1-α* and *dsx1-β* begin to accumulate exclusively in male embryos as early as 18 hours after ovulation. Dimorphism until the young daphniids were released from the mother’s brood chamber (3-day-old) has been reported, but it is unclear when male-specific expression begins and whether this pattern is maintained in the adulthood. To confirm this finding, we performed quantitative PCR using total cDNA obtained from early embryos up to adult daphniids (Fig. 1). As a result, the *dsx1-α* transcript became abundant in males around the gastrulation stage, at 9 h post-ovulation (hpo). Consequently, elevated expressions of both *dsx1-α* and *dsx1-β* were detected in males, which were steadily maintained during embryogenesis (Fig. 1A) and throughout the maturation period (from instar 1 to instar 5), without any prominent decrease or fluctuation patterns (Fig. 1B). Towards the first wave of oogenesis during instar 4, there was a slight increase in *dsx1-β* expression, probably due to the deposition of this mRNA in the developing oocytes.

### Generation of the hemizygous *dsx1*^+^*/mCherry-dsx1*^*−*^ reporter strain

**Figure 2:**
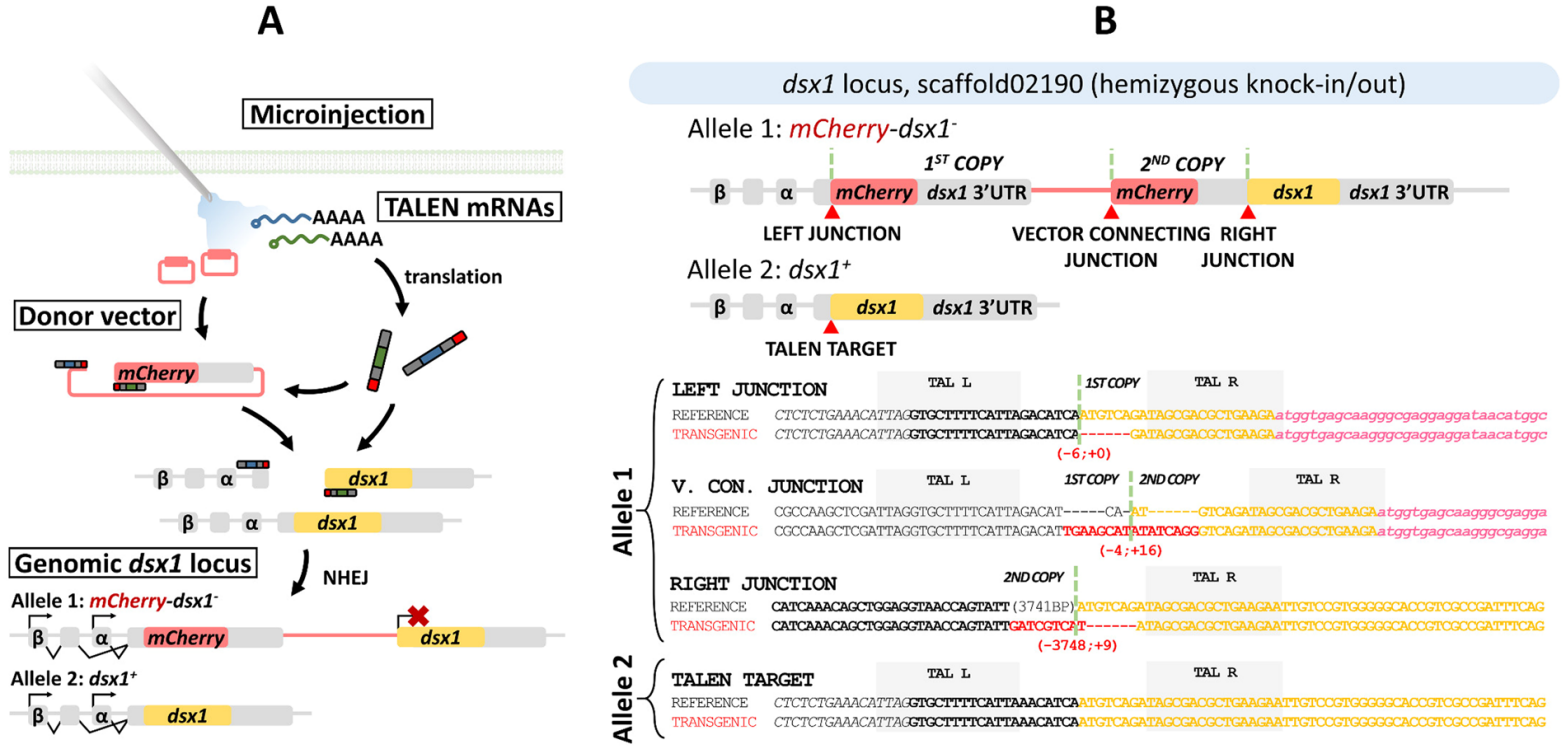
Generation of the *dsx1* reporter strain. (**A**) Knock-in approach. See description in the main text. (**B**) Genotype of the obtained *dsx1* reporter strain. At the *dsx1* locus, targeted hemizygous knock-in of two tandem copies of the reporter cassette was achieved, leading to the silencing of *dsx1* on this allele. Therefore, the genotype at this locus could be depicted as *dsx1* ^+^ */mCherry-dsx1* ^*−*^. Genomic sequences of regions susceptible to in-del mutation after knock-in (left and right junctions, connecting junction of the two donor copies, and TALEN target sequence) are shown. For the reference sequences, an NHEJ was assumed to occur without causing any in-del mutations. Scaffold and contig information were obtained from the “2015 March *Daphnia magna* Gene-ome early release dataset” (http://arthropods.eugenes.org/EvidentialGene/daphnia/daphnia_magna/). Images are not to scale.

To create an *in vivo* reporter that can mimic the expression of *dsx1*, we used a TALEN-based genome editing toolset previously established in *D*. *magna* ^19,21^. A pair of TALENs was designed to induce double-strand breaks immediately before the start codon of *dsx1* open reading frame (ORF) (Supplementary Fig. S1). In the donor vector, a 46-bp sequence including the TALEN-targeting site was placed upstream of the *mCherry* gene (Supplementary Fig. S2) to enable the vector and genome to be simultaneously cleaved by TALENs inside the cells. We expected that a copy of the donor vector would be integrated into one of the two *dsx1* alleles *via* the endogenous NHEJ machinery, while the other allele would remain intact, allowing both *mCherry* and *dsx1* to be expressed under the control of the endogenous *dsx1* alternative promoters, *α* and *β*, both of which share the same *dsx1* ORF (Fig. 2A). Furthermore, to ensure that *dsx1* expression was accurately reported, the full-length 3′ UTR of the *dsx1* gene was linked to the *mCherry* ORF.

Target cleavage activity of the TALENs was first tested *in vivo*, and the efficiency of somatic knock-out was estimated at approximately 50% (Supplementary Fig. S3), which was similar to that reported for eyeless-targeting TALENs previously used for knock-in experiments in this species^21^. We used a transgenic *D*. *magna* strain, containing an *EF1α-1::h2b-gfp* transgene, as the host strain in knock-in experiments in order to map the internal structure of the animal^25^. Eighty-three eggs were subjected to microinjections, of which 18 survived to adulthood. Among these, two produced offspring harbouring the reporter construct in the genome (Supplementary Fig. S4). Two transgenic lines were established from those two founders.

Genotyping analyses showed that, in one of the two transgenic lines, hereby referred to as the *dsx1* reporter strain, one allele did not carry any mutations, while another contained the desired knock-in construct (Fig. 2B). In that line, two tandem copies of the donor vector were integrated into the *dsx1* locus with the right junction containing a large deletion (−3748; +9). The second copy of the *mCherry* gene would be silenced as there was no possible promoter. Therefore, this *dsx1* reporter strain, whose genotype can be designated as *dsx1* ^+^ */mCherry-dsx1* ^*−*^, was selected for further characterisation.

### Hemizygous *dsx1*^+^*/mCherry-dsx1*^*−*^ males and females are sexually functional

**Figure 3:**
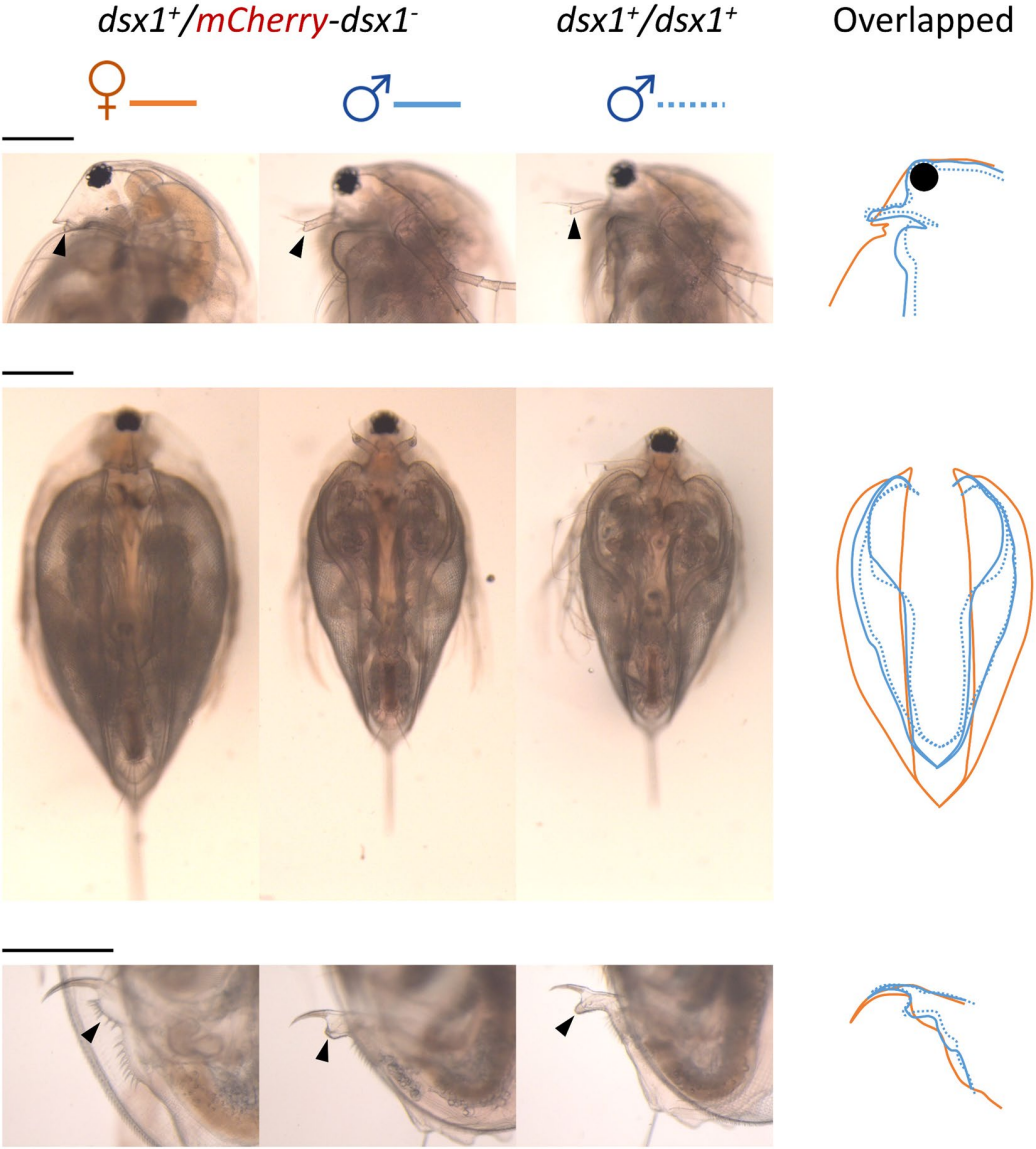
Morphology of hemizygous *dsx1* knock-out *D*. *magna*. Adult (21-day-old) male and female individuals of the *dsx1* ^+^ */mCherry-dsx1* ^*−*^ genotype showed typical morphology. From top to bottom: side view of the anterior region showing the rostrum and first pair of antennae; full body ventral view showing the edge of the large carapace; and side view of the posterior region showing the genital. For comparison, the morphology of males from the background strain (*dsx1* ^+^ */dsx1* ^+^) is included. Black arrow head: first antenna. White arrow head: genitalia. Tracing lines on the ‘overlap’ panels depict the outline of the body. Scale bars = 0.5 mm.

To confirm whether the *dsx1* hemizygous knock-out genotype has any adverse effect on the reproductive function of males and females, we observed the morphology of several external sexual characteristics in adult daphniids. Comparisons were made for the three most profound sex-specific traits: rostrum–first antennae, carapace edge, and genitals (Fig. 3). Both male and female individuals of the *dsx1* reporter strain exhibited a typical phenotype. Males were smaller in size, with an angular rostrum and a prolonged first pair of antennae, a curve-opened carapace, and a pointed penis. Females had a bulky rostrum with reduced antennae, the genitals were rounded, and the carapace edge was less exposing. However, when the structures of the sex-specific traits in the male *dsx1* reporters were overlaid with those of wild-type animals, a minor feminisation effect was detected. In detail, when comparing adults of the same age (Fig. 3), the first antennae of the hemizygous males were thicker and slightly shorter than those of the wild-type ones. Their body length was also considerably greater, with the edge of the carapace curved into a less angular shape. In particular, their penises could not achieve the typical acute outline and instead, remained rather blunt. The morphology of male *dsx1* reporters was different from that of a wild-type male, displaying a slight shift towards a feminine appearance.

**Figure 4:**
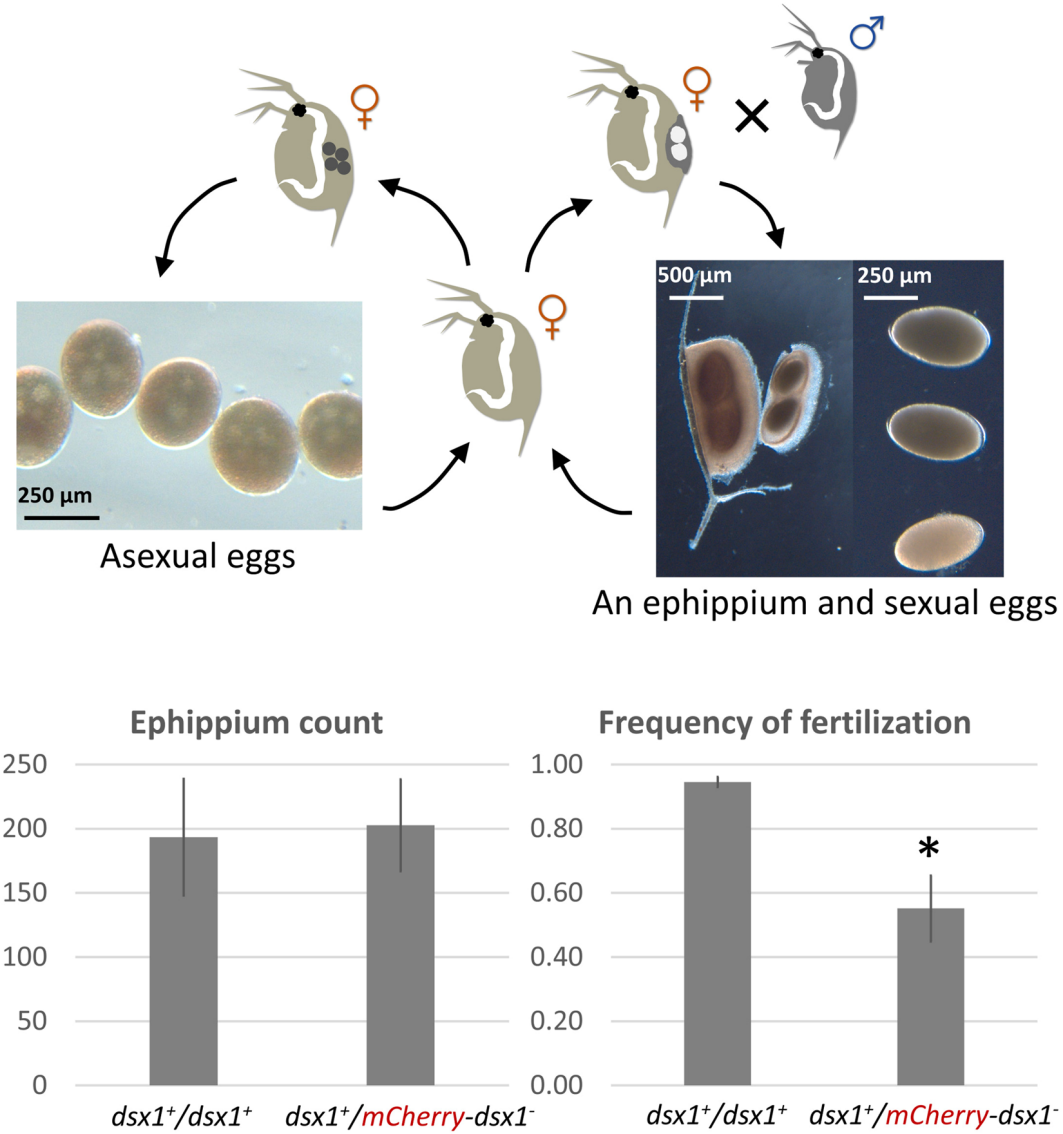
Reproductive ability of hemizygous *dsx1* knock-out male. Male and female individuals of the *dsx1* reporter strain could reproduce normally. Typically, wild-type females clone themselves under favourable living conditions by producing asexual eggs, while under unfavourable conditions, females ovulate sexual eggs that need to be fertilised and deposited into protective structures called ephippia. In this reproduction test, daphniid populations were subjected to stressful conditions to induce sexual reproduction. For each strain (*dsx1* reporter and wild-type), 175 females and 25 males were cultured, in triplicate, for 4 weeks, and ephippia were collected at the end of cultivation. Total ephippia were counted and the frequency of fertilisation was calculated (see Methods) separately for each population. Graphs show mean values from the triplicates. Error bars indicate standard deviation. *p < 0.05 (*t*-test).

Similarly, in a reproduction test involving a cross between male and female *dsx1* reporters, the next generation was successfully obtained, indicating that females could produce sexual eggs and males could mate and fertilise normally (Fig. 4). The *dsx1* reporter strain had the same ability as the wild-type to produce a protective case called an ephippium, where two sexual eggs are deposited, suggesting that the female’s ability to enter a sexual mode under stressful conditions was comparable between the *dsx1* reporter and the wild-type. However, in the male *dsx1* reporters, the frequency of successfully fertilised sexual eggs was reduced by 1.7-fold, implying a feminisation effect of the hemizygous *dsx1* locus.

We further quantified the level of *dsx1* expression in our established *dsx1* reporter line to determine whether one less copy of *dsx1* would affect the overall transcription of this gene (Supplementary Fig. S5). Consistent with the slight feminisation of male phenotypes observed, *dsx1* expression in 0-, 24-, 48-, and 72-h embryos was reduced by 2.2-, 1.4-, 2.8-, and 2.6-fold, respectively, compared to that observed in the male wild-type *dsx1* ^+^ */dsx1* ^+^. The sexual dimorphic pattern remained consistent with that observed in the wild-type.

### *mCherry* expression in the *dsx1*^+^*/mCherry-dsx1*^*-*^ strain is consistent with *dsx1* expression

**Figure 5:**
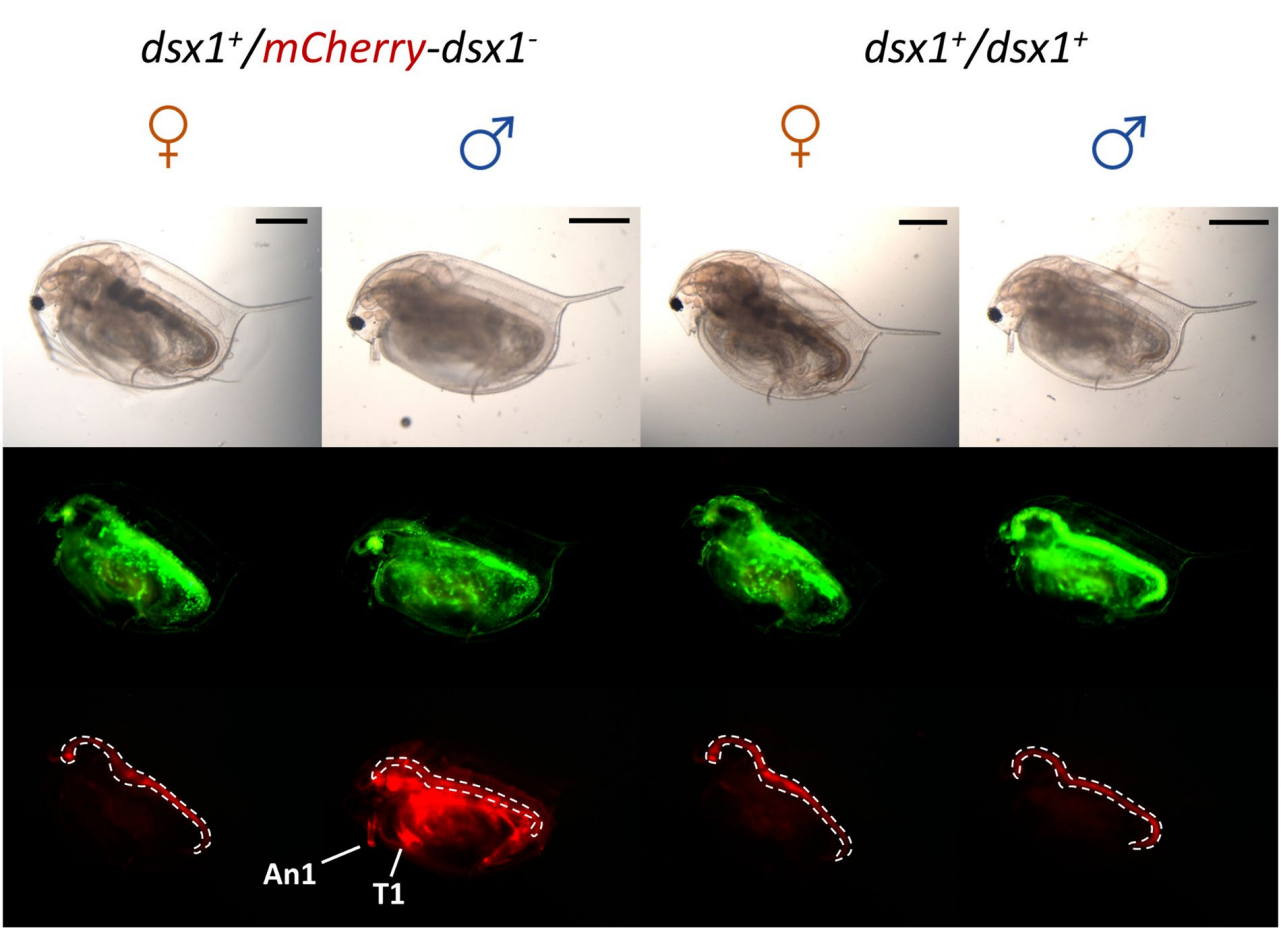
Fluorescence of the *dsx1* ^+^ */mCherry-dsx1* ^−^ reporter strain. Nine-day-old fully matured daphniids were observed under bright-field (top row), GFP filter (middle row), and mCherry filter (bottom row). Ubiquitous green fluorescence is caused by the *EF1α-1::h2b-gfp* background genotype. In all cases, the red signal from the guts (dotted lines) represents the autofluorescence of *Chlorella*, the main food used in daphniid cultivation. An1: first antennae. T1: first thoracic legs. Scale bars = 0.5 mm. Pictures were taken under the same camera settings.

**Figure 6:**
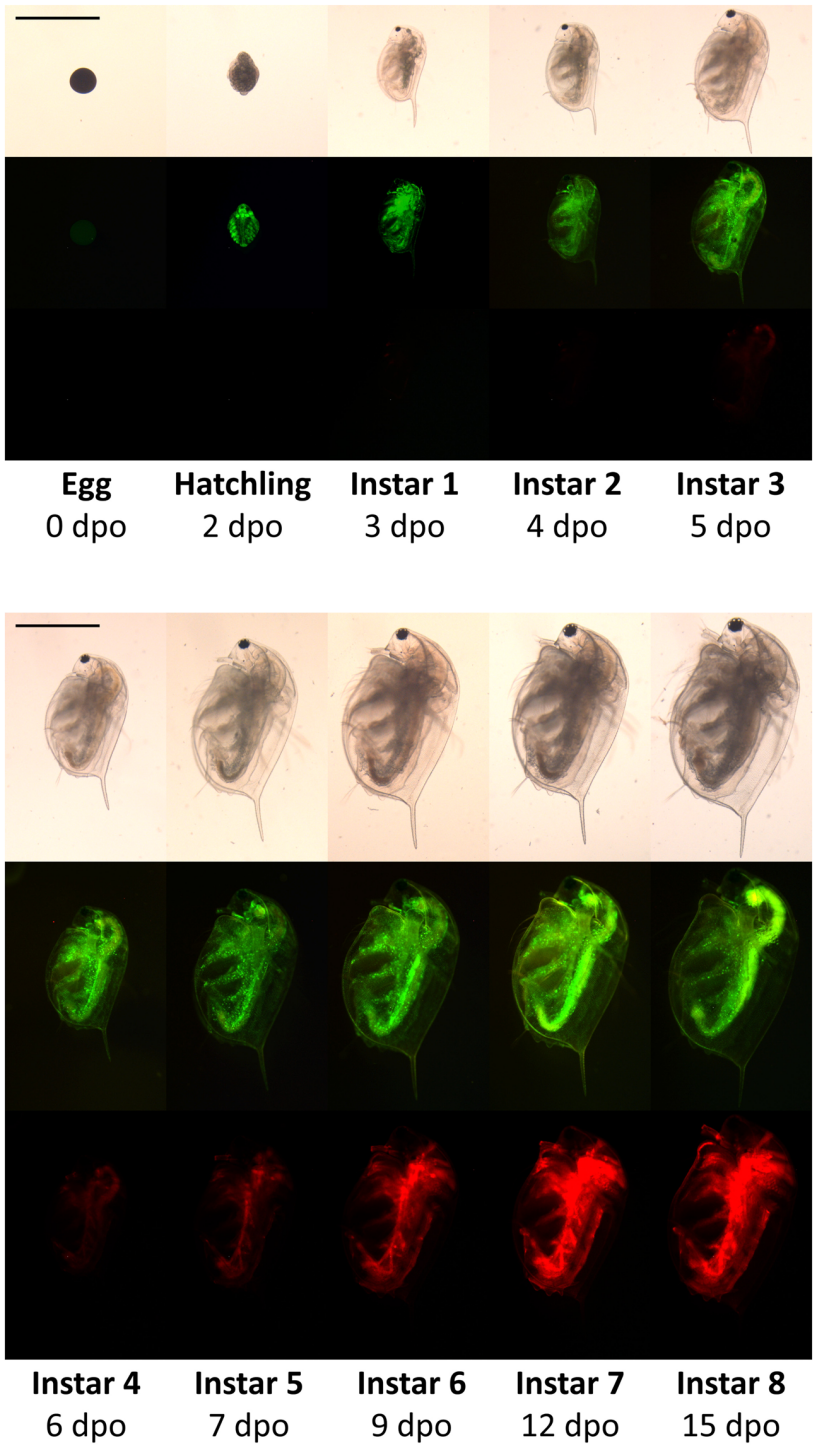
Temporal change of fluorescence of the *dsx1* reporter *Daphnia*. Pictures of the male reporter *Daphnia* from different development stages were recorded. The first swimming juvenile released from the mother’s brood chamber (usually 3 days after ovulation) is designated as instar 1. Every time the animal moults, it moves to the next instar stage. Typically, after 4–6 moults, daphniids become matured adults. Scale bar = 0.5 mm. Pictures were taken using the same camera settings. dpo: day(s) post-ovulation.

Phenotypic characterisation, focusing on *mCherry* behaviour in the *dsx1* reporter strain, was carried out. First, by observing adult daphniids, we confirmed that the mCherry signal was male-specific, with no specific fluorescence signal observed in the females (Fig. 5). The first pair of antennae and thoracic legs showed particularly high intensity of mCherry, similar to that previously reported for *dsx1* ^24^. Next, the spatiotemporal intensity of mCherry expression in male animals was determined by periodically capturing images each time the animal moulted. As the animal gradually matured throughout development, GFP intensity remained stable, whereas mCherry intensity increased steadily (Fig. 6). In the early stages, red fluorescence was weak and restricted to certain locations, as described later. With maturation, fluorescence intensity increased and expanded to a wider range of tissues.

According to a previous publication^24^, and to the qPCR data generated in our study (Fig. 1), the behaviour of the *mCherry* reporter in the *dsx1* ^+^ */mCherry-dsx1* ^*−*^ strain was consistent with that of the wild-type *dsx1*. Additionally, we characterised the transcription level of *mCherry* using qPCR, and could observe *dsx1* sexual dimorphism (Supplementary Fig. S5). Therefore, we concluded that in the generated *dsx1* reporter strain, *mCherry* could recapitulate the activity of *dsx1* and therefore, could be used as a visible marker of *dsx1* expression in cells.

### Expression of *dsx1* is time- and tissue-specifically synced with maturation events

**Figure 7:**
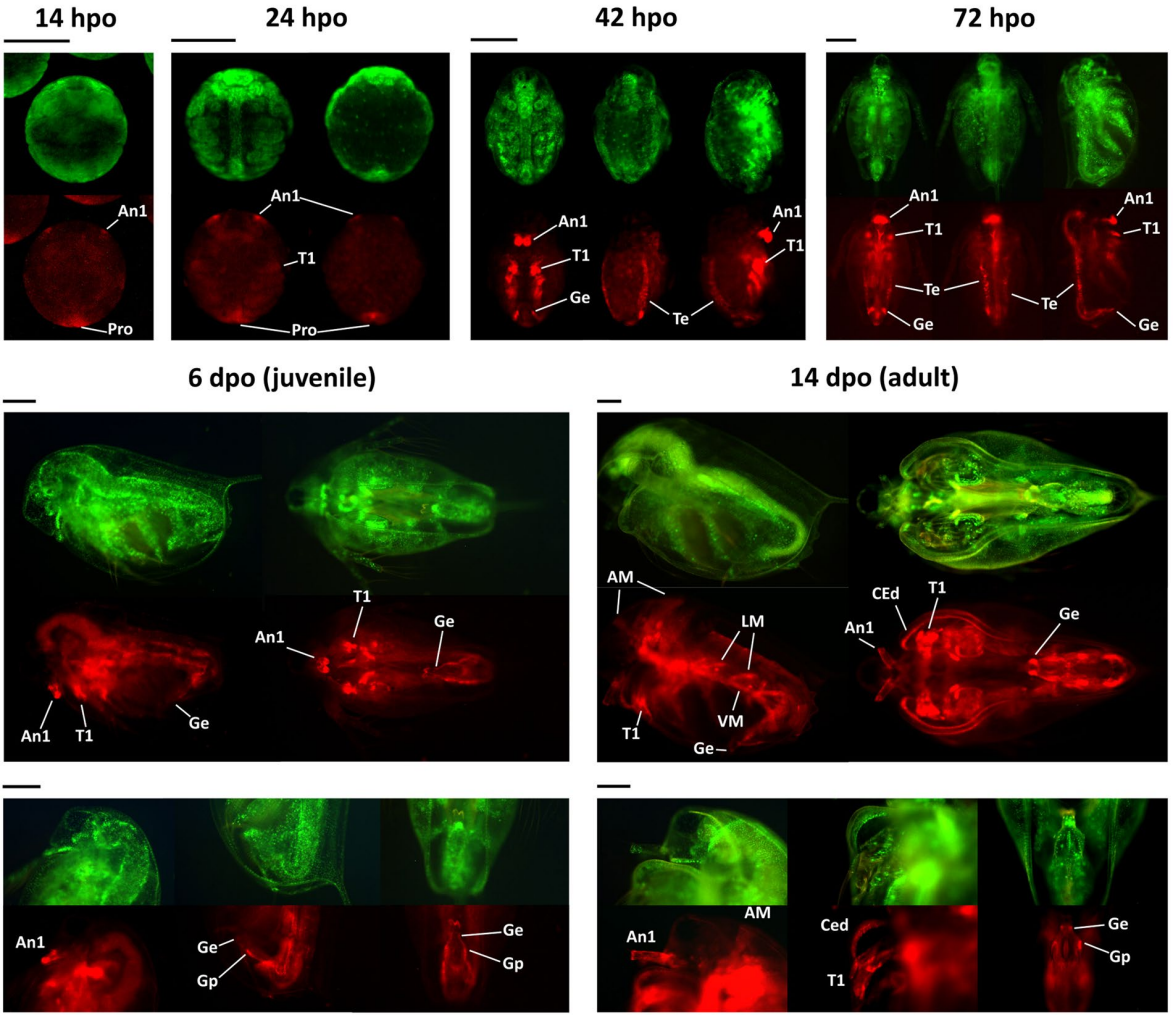
Spatially and temporally specific expressions of *mCherry* in the *dsx1* reporter strain. All embryos and daphniids shown in this figure are males of the *dsx1* ^+^ */mCherry-dsx1* ^*−*^ strain. hpo: hours post-ovulation. dpo: days post-ovulation. For the 14 hpo panel: ventral view. For the 24 hpo panel: ventral view (left) and dorsal view (right). For the 42 hpo and 72 hpo panels: ventral view (left), dorsal view (middle), and side view with the ventral side facing right (right). A1: first antenna, T1: first thoracic leg, Pro: proctodeum, Ge: genital (i.e. penis in this case), Te: testis, AM: antennal muscle, LM: lateral muscle, VM: ventral muscle, CEd: carapace edge, Gp: gonopore. Scale bars = 200 μm.

*mCherry* expression was observed in embryos of the *dsx1* reporter strain at 14-, 24-, 42-h post-ovulation (hpo), juveniles at 72 hpo and 6 days post-ovulation (dpo), and adults at 14 dpo (Fig. 7). Based on our observations, major developmental stages corresponding to the presence of mCherry in male animals can be described as follows:

(i) Right before body segmentation occurred (14 hpo), the mCherry signal was obvious in (1) the rudiment first pair of antennae and (2) the posterior zone around the proctodeum. At 24 hpo, embryos started to exhibit strong mCherry fluorescence in a pair of first thoracic legs and a weaker signal in the other thoracic legs.
(ii) In the later embryos (42 hpo), mCherry signal was primarily found in (1) a pair of first antennae, (2) pairs of thoracic legs, and (3) the testes and spermiduct-genitalia system. At 72 hpo, among the thoracic legs, an intense signal was maintained only in the first thoracic legs.
(iii) Following the onset of maturation, the signal in the testes gradually weakened, and had disappeared at 6 dpo. Conversely, a collection of other male-specific structures began to strongly express mCherry. In adult males (14 dpo), the signal was particularly intense in (1) a pair of first antennae, (2) a pair of first thoracic legs (which had already developed into copulation hooks), (3) the carapace edges below the helmet, (4) the skeletal muscle system, and (5) penises and gonopores.

Interestingly, the timing of *dsx1* activation in male-specific structures corresponded to that of their maturation. Until instar 4 (6 dpo), except for the elongated first antennae and the gonad, there was no clear dimorphism between males and females (Figs 6, 6 dpo). From instar 5, there was a surge in *dsx1* activity, as shown by the rapid accumulation of mCherry into several male-specific structures, including the carapace edge below the helmet, the tip of the penis, and the skeletal muscle (Fig. 6, 7, 14 dpo). Clear transformation of the young juveniles into matured males was observed, in which the carapace edge began to curve at the mCherry-expressing section to create an opening, and the tip of the penis was raised to form an acute shape (Figs 6, 7, 14 dpo). Consistent with this observation, the increased expression of *dsx1-α* was also detected using qPCR analysis (Fig. 1). These results suggest that there is a time- and site-specific role of *dsx1*. This gene is only recruited when and where it is needed, and at that moment, it begins to dictate the sexual differentiation of that location.

### *dsx1* is active in early progenitor cell clutches

**Figure 8:**
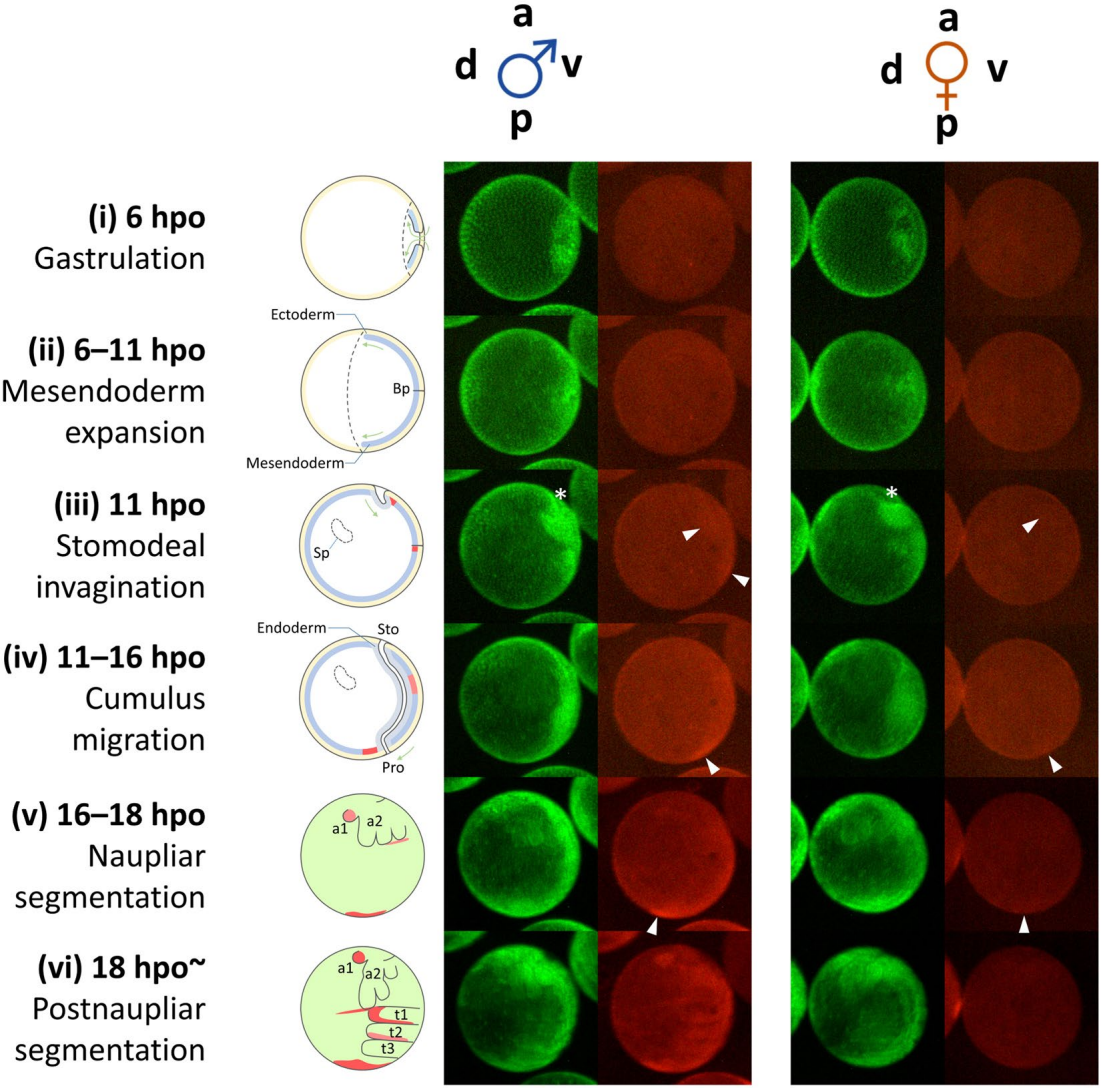
Dynamic progression of *dsx1-*expressing cells during early embryogenesis. This snapshot series of two males and one female of the *dsx1* reporter strain was extracted from Supplementary Movie S1, showing major events from gastrulation until thoracic segmentation. From top to bottom are frame 39, 50, 60, 69, 102, and 137 from the movie. Schematic diagrams on the right illustrate simple anatomy of the embryos at the corresponding time points. The diagrams from stage (i) to (iv) show images of embryonic sections. The diagrams (v) and (vi) show superficial views of embryos. a: anterior, p: posterior, v- ventral, d: dorsal, Bp: blastopore, Sp: Scheitelplatten, Sto: stomodeum, Pro: proctodeum, t1–3: thoracic segments, a1: first antenna, a2: second antenna, hpo: hours post ovulation. White arrow heads indicate mCherry-expressing cell clusters that migrate in an anterior-to-posterior direction. An asterisk indicates the site of invagination.

To understand which cell population first shows *dsx1* expression, a time-lapse observation of the early stages of development was performed in males of the *dsx1* ^+^/*mCherry-dsx1* ^−^ reporter strain (Fig. 8, Supplementary Movie S1–S3, Supplementary Figs S6–S8). Observation of the H2B-GFP signal allowed us to distinguish six different early embryonic stages: (i) gastrulation, (ii) mesendoderm expansion, (iii) stomodeal invagination, (iv) cumulus migration, (v) naupliar segmentation, and (vi) postnaupliar segmentation. We identified two previously undescribed stages, (iii) and (iv). At 11 hpo, a second gastrulation, termed stomodeal invagination, occurred between the blastopore and the anterior end, forming the origin of the mouth. From 11 to 16 hpo, the internalised cells, named “cumulus”, migrated along a midline, situated on the ventral region, towards the caudal side (Fig. 8, Supplementary Movie S3).

Although no particular signal was detected until stage (ii), we found that mCherry was first localised into the cell cumulus and appeared around the site of invagination. At the same time, a second signal could also be found around the blastopore. As development proceeded, together with the cumulus, the mCherry-expressing cell cluster migrated towards the blastopore and gradually became less localised. In parallel, the second mCherry-expressing cell cluster moved downwards until reaching the posterior. It was not clear whether those two mCherry-expressing cell clusters merged, but the closer they got to the posterior region, the stronger the red fluorescence became. At stage (v), as the buds of the first antennae emerged, the mCherry signal also intensified in this region. Along the border of the head and body segments, a significant signal was also detected. At stage (vi), thoracic segmentation began, and the five segments appeared in order from anterior to posterior. The first segment, which would subsequently develop into the male-specific copulation hook in adults, strongly expressed mCherry. Fluorescence was also detected in other segments, but at a lower level. Just before hatching, embryonic regions exhibiting reporter gene activity were: (1) around the proctodeum, (2) the first antennae, (3) the thoracic segments, especially the first one, and (4) the head-body border.

The same procedure was also performed for *dsx1* ^+^ */mCherry-dsx1* ^*−*^ females of this reporter strain (Fig. 8, Supplementary Movie S3). Very similar mCherry patterns were observed prior to segmentation. A signal was detected in the migrating cumulus and blastopore/proctodeum area, but it was much weaker in the embryos than that observed in males. From stage (v), the signal neither intensified nor appeared elsewhere in females. These results were consistent with those obtained using qPCR (Fig. 1), except for the expression patterns at 6 hpo, when *dsx1* was expressed in both male and female embryos. This contradiction probably resulted from the rather weak expression level of the *mCherry* reporter, which might be indistinguishable from the noise generated by the yolk protein.

## Discussion

Although numerous studies have widely investigated the development of sex-specific traits, our understanding of this process is limited to model organisms such as mouse, *Drosophila*, and *Caenorhabditis elegans*. To better understand both the commonality and evolution of sex-specific trait development in animals, we analysed the spatiotemporal expression of a DM-domain gene, *dsx1*, from the newly emerging crustacean genetic model, *D*. *magna* ^24^, because of its ancestral position within the Pancrustacean clade^16^ and unique sex determination system, whereby environmental signals stimulate asexual male production^17^. In the present study, by using a TALEN-mediated genetic approach, we generated a transgenic *Daphnia* that allows the visualisation of *dsx1* expression based on mCherry fluorescence. This *dsx1* reporter strain highlights the strict temporal and spatial synchronisation of *dsx1* expression and the development of sexually dimorphic traits. From our data, we discuss two features of *dsx1* expression patterns, i) expression and its maintenance in male-specific tissues and ii) early embryonic expression.

First, *dsx1* expression is spatiotemporally regulated and maintained in male-specific traits. This feature is consistent with the DM-domain genes found in other studied organisms such as fruit fly^8–11^ and mouse^13,14^. In mammals, *Dmrt1* (a mammalian DM-domain gene) is upregulated during late testicular development, and its expression in the testis is consistently maintained by a positive regulation loop involving *Sox9*, *Fgf9*, and *Ptgdr* until adulthood^26^. In *Drosophila*, in addition to sex-specific splicing, *dsx* is regulated at the transcriptional level and exhibits ongoing expression for the maintenance of sexual traits in a small subset of cells located in the dimorphic tissues^8,27^. Therefore, our study supports a common idea: sex-determination is restricted temporally and spatially in the ability to recruit a DM-domain gene; once activated, this gene subsequently takes over the role of sex maintenance.

Second, male-specific *dsx1* expression begins in a specific cell cluster of early embryos. The specific cell population “cumulus” first expresses mCherry at the stomodeal invagination stage, and it subsequently migrates towards ventral regions, from rostral to caudal, along a midline. The functions of similar migrating cell populations have already been characterised in other animals. In chicken, a mass of cells called “Hensen’s Node” shows similar movements during gastrulation and neurulation^28^. In spider, a cell mass named “cumulus” has also been reported to appear at the central blastopore and migrate centrifugally^29^. Importantly, both migrating cell masses function as primary organisers and secrete common molecules such as BMP/Dpp for axial pattern formation^30^. This suggests that the primary organiser is sexually plastic in *D*. *magna*. Thereafter, the cumulus was located and started to show stronger fluorescence in the posterior region, or posterior growth zone. Posterior growth and segmentation are known to be accomplished by the supply of progenitor cells from the back end of the embryo^31^. *dsx1-*expressing progenitor cells would be distributed from the posterior growth zone in early embryos across the body during axial elongation. Considering the phylogenetic position of the cladoceran crustacean *Daphnia* ^16^, expression patterns of the DM-domain gene during the basal body formation process might be conserved in this clade. Because this is, to our knowledge, the first time-lapse imaging-based tracking of DM-domain gene expression in early embryos, further comparative expression analyses in other organisms will be necessary to determine the similarity of DM-domain gene expression in early embryos.

This work demonstrates *dsx1* expression patterns in space and time using the knock-in reporter gene *mCherry*. *dsx1* in this species is clearly involved in male-specific trait expression. Its expression begins in cell populations that may control developmental patterning. We anticipate that this work will contribute to the further understanding of not only sexually dimorphic trait development, but also environmental sex-determining mechanisms in the future.

## Methods

### *Daphnia* strains and culture conditions

A previously established transgenic strain of *D*. *magna* carrying two tandem copies of an EF1α-1::H2B-GFP construct^25^, collectively referred to as wild-type, was used in the present study. In this strain, the strong and ubiquitous expression of a H2B-EGFP fusion protein is driven by the *D*. *magna* EF1α-1 promoter/UTR cassette, resulting in a permanent green fluorescent signal that can be observed in cell nuclei throughout the animal body. This genetic background provides an easy definition of the animal’s anatomy at every stage of development.

For regular cultivation, daphniids were maintained as populations containing 80 females in 5 L ADaM^32^ medium. Each population was fed daily with 1.12 × 10^9^ *Chlorella vulgaris* cells, and new-born juveniles were removed regularly. The ambient temperature was kept at 22–24 °C, and the light/dark photoperiod was fixed at 16/8 h. For daphniids observed using fluorescence microscopy, the algae were replaced by an equivalent amount of dry yeast to avoid autofluorescence.

### Designing TALENs

The *dsx1* locus in the host strain was previously sequenced independently in our laboratory (unpublished data). This sequence was used as the query sequence in the web-based tool TAL Effector Nucleotide Targeter (TALE-NT) 2.0^33^ using the following parameters: spacer length = 15–20, repeat array length = 15–20, G substitute = NN, Streubel *et al*. guidelines = On, upstream base = T only. A design potentially targeting immediately adjacent to the start codon of Dsx1 ORF was chosen.

### Construction of TALEN expression vectors

In order to assemble the full coding sequence of each TALEN (left and right) into a vector suitable for *in vitro* transcription, the Golden Gate method was used as previously described^34^. Briefly, at first, the RVD units were assembled onto the GoldyTALEN backbone, resulting in the generation of pT3TS-dsx1start-TALEN-left and -right. Subsequently, from these vectors, the DNA-binding domains were swapped with those from pCS-Dmavas-dsr-TALEN-left-ELD and -right-KKR, using a pair of restriction enzymes (XbaI and BsaBI)^19^, leading to the final construction of pCS-Dmavas-dsx1start-TALEN-left-ELD and -right-KKR expression vectors.

### *In vitro* transcription

To generate linear templates for *in vitro* transcription, the two TALEN expression vectors were first digested with Acc65I, and then subjected to purification using a QIAquick PCR purification kit (QIAGEN GmbH, Hilden, Germany). With these templates, capped-transcription was performed using the mMessage mMachine SP6 kit (Life Technologies, California, USA), followed by a tailing reaction using a Poly(A) Tailing kit (Life Technologies). Further, the products underwent a three-step purification using miniQuick Spin RNA columns (Roche Diagnostics GmbH, Mannheim, Germany), phenol/chloroform purification, and ethanol purification, before being dissolved in DNase/RNase-free water (Life Technologies).

### Construction of donor vector

The donor vector consisted of three main elements: i) a recognition sequence for the designed TALEN pair (48 bp), ii) a full coding sequence of the red fluorescent protein-encoding gene *mCherry* (711 bp), and iii) a full-length sequence of *dsx1* 3′ UTR cloned directly from the last exon of the host animal (2,458 bp). These elements were seamlessly assembled in the respective order onto the pRN3 backbone using the In-Fusion® HD Cloning Kit (Clontech), resulting in a 6,057-bp plasmid.

### Microinjection

To introduce foreign materials into young embryos of *D*. *magna*, we followed a previously established microinjection-based protocol^35^. Briefly, freshly ovulated eggs from 2- to 4-week-old *Daphnia* mothers were retrieved. The eggs were then incubated in ice-cold M4-sucrose solution, and microinjected using home-forged glass needles on a specialised platform within 1 hour. The injection solution contained 500 ng/μL of each TALEN mRNA with or without 50 ng/μL of donor vector. Approximately 0.2 nL of the solution was injected into each egg. The injected eggs were then incubated in M4-sucrose for at least 3 days at 22–24 °C before being transferred to ADaM medium.

### TALENs functionality test

TALENs were injected into *D*. *magna* embryos without the presence of the donor vector. After 72 h, each injected animal was collected separately and total genomic DNA was extracted. In-del mutations at the target site of TALENs were confirmed using a pair of primers (*Dsx1*_start_left and *Dsx1*_start_right, Supplementary Table S1) that amplifies a 186-bp region around the wild-type *Dsx1* start codon. PCR products were separated on 20% polyacrylamide gel and stained with SYBR Green I.

### Screening for G0 candidates

To screen for G0 candidates obtained by co-injection of TALEN mRNAs and the donor vector, all G0 daphniids that could survive until reproduction age were cultured separately, and germline transmission of the donor vector was analysed using G1 offspring. Three PCR-based tests were carried out: i) the primer pair *mCherry*_check_F and *mCherry*_check_R, which amplified a 184-bp region within the *mCherry* CDS, was used to confirm the presence of donor vector in the genome, ii) the primer pair *Dsx1*_start_left and *mCherry*_check_R, which amplified a 729-bp region spanning the left junction of the expected genotype, was used to confirm the integration of the donor vector into the target site in the correct direction, and iii) the primer pair *Dsx1*_start_left and *Dsx1*_start_right, which amplified a 186-bp region around the *dsx1* start codon, was used to confirm the integrity of the other allele. G0s that were positive for all three tests were then selected as the founder animals. Information on these primers can be found in Supplementary Table S1.

### Genotyping of the knock-in candidates

At first, quantitative PCR was used to determine the copy numbers of donor vectors integrated into the host genome. The CDS of *mCherry* was targeted using the primer pair *mCherry*_qPCR_F and *mCherry*_qPCR_R (product size = 112 bp). The housekeeping gene encoding ribosomal protein *L32*, which has two copies in the *D*. *magna* genome, was used as the reference, and was targeted by the primer pair DmagRP*L32*-realtime-5 and DmagRP*L32*-realtime-3 (product size = 67 bp).

Next, regions susceptive to in-del mutations were amplified using specific primers: Seq_*Dsx1*Start_L and Seq_*Dsx1*Start_R for the non-inserted *Dsx1* allele (TALEN target), Seq_mChVector_R (head) and Seq_mChVector_L (tail) for the *Dsx1* promoter–vector junction (left junction), right_junc_6kb_L and right_junc_3kb_alt_R for the vector–*Dsx1* ORF junction (right junction), and vect_connect_L and vect_connect_R for the vector–vector junction (vector connecting junction). The obtained fragments were cloned using the Zero Blunt® TOPO® PCR Cloning Kit (Thermo Fisher Scientific) and ultimately sequenced. Information on these primers can be found in Supplementary Table S1.

### Production of male daphniids and resting eggs

To induce the production of males from mother daphniids, a previously described method was used^36^. In short, 2- to 3 -week-old females were treated with 1 μg/L Fenoxycarb (Wako Pure Chemicals, Osaka, Japan) so that oocytes at sensitive stages (50–56 h of ovarian development) could be exposed to the chemical. All individuals of the oocyte clutch would later develop into males.

To induce resting egg production, males and females were kept together in a 1:7 ratio under a high population density and starving conditions. In detail, 25 males and 175 females were cultured in the same beaker containing 1 L ADaM, and fed with 4.2 × 10^8^ *Chlorella* cells/mL once a day. Females were expected to produce ephippia after 1 or 2 weeks.

From each beaker, all collected ephippia were opened to retrieve the resting eggs inside. Further, data were recorded for total ephippium count and total egg count. The frequency of fertilisation = total egg count/(total ephippium count × 2).

### Quantitative PCR

Total RNA was extracted from sample triplicates and subjected to cDNA synthesis using random hexamers (Invitrogen). SYBR^®^ GreenER™ qPCR SuperMix Universal Kit (Invitrogen) was used for qPCR, and reactions were conducted using a Mx3005 P Real-Time PCR System (Agilent Technologies, CA, USA). To amplify the common CDS and specific 5′ UTRs of the two *dsx1* transcripts, previously described primers^24^ were used. To amplify the *mCherry* CDS, the *mCherry*_qPCR_F and *mCherry*_qPCR_R pair **(**Supplementary Table S1 **)** was used. To amplify the housekeeping *L32* gene, the DmagRP*L32*-realtime-5 and DmagRP*L32*-realtime-3 pair **(**Supplementary Table S1 **)** was used.

### Data availability statement

All data generated or analysed during this study are included in this published article (and its Supplementary Information files).

## Electronic supplementary material

Supplementary information

Supplementary Movie S1

Supplementary Movie S2

Supplementary Movie S3
