# Supplementary material for: Mapping the expression of the sex determining factor Doublesex1 in Daphnia magna using a knock-in reporter
Source: Sci Rep. 2017 Nov 2;7:13521. doi: 10.1038/s41598-017-13730-4 (PMC5668254; doi:10.1038/s41598-017-13730-4)
Supplement: Supplementary file 1 — Supplementary information [file 41598_2017_13730_MOESM1_ESM.pdf]

SUPPLEMENTARY DATA

Mapping the expression of the sex determining factor *Doublesex1* in *Daphnia magna* using a knock-in reporter

Nong Dang Quang, Nur Syafiqah Mohamad Ishak, Tomoaki Matsuura, Yasuhiko Kato*, Hajime Watanabe

* Corresponding author: Yasuhiko Kato (kato_yasuhiko@bio.eng.osaka-u.ac.jp)

Supplementary Table S1. Primer list. All primers used in this study were synthesized by FASMAC (Tokyo, Japan).

**Primer name**	**5' → 3' sequence**
Dsx1_start_left	CGGCTTATCTAAAATGTCGGCCC
Dsx1_start_right	CCCACGGACAATTCTTCAGCG
mCherry_check_F	CTACAAGGTGAAGCTGCGCG
mCherry_check_R	GGTGGTCTTGACCTCAGCGT
Seq_Dsx1Start_L	GAAGGGAAAGCTTCGTACCGA
Seq_Dsx1Start_R	GTGATTGTTGACGCCGAGGA
Seq_mChVector_R(head)	GAAGTTCATCACGCGCTCCC
Seq_mChVector_L(tail)	TTTGTGATGCTCGTCAGGGG
vect_connect_L	CAGGTATCCGGTAAGCGGCA
vect_connect_R	GGTGGTCTTGACCTCAGCGT
right_junc_6kb_L	GAAGGAGAACGTACTGTATTATCGAGGC
right_junc_3kb_alt_R	ATAAAGTTTGGTGTAGGGGAGGATGAGG
mCherry_qPCR_F	CTACGACGCTGAGGTCAAGAC
mCherry_qPCR_R	GGTGTAGTCCTCGTTGTGGG
DmagRPL32-realtime-5	GACCAAAGGGTATTGACAACAGA
DmagRPL32-realtime-3	CCAACTTTTGGCATAAGGTACTG

A. Query sequence

5’-TGAAAAGAATCTAAACCCAATTGTTTTTTTGTTTTCTTTTCTCTCTGAAACATTAGGTGCTTTTCATTAGAC
ATCAATGTCAGATAGCGACGCTGAAGAATTGTCCGTGGGGGCACCGTCGCCGATTTCAGCTAAAGAGCGC-3’

(= exon, = ORF)

B. The chosen design of TALEN pair

options_used:array_min = 15, array_max = 20, spacer_min = 15, spacer_max = 20, upstream_base = T									
**Sequence Name**	**Cut Site**	**TAL1 start**	**TAL2 start**	**TAL1 length**	**TAL2 length**	**Spacer length**	**Spacer range**	**Unique RE sites in spacer**	**% RVDs HD or NN/NH**
dsx1	120	97	142	16	15	15	113-127	DrdI:GACNNNN NNGTC	42
**TAL1 RVDs**	NG NI NN NN NG NN HD NG NG NG NG HD NI NG NG NI								
**TAL2 RVDs**	NG HD NG NG HD NI NN HD NN NG HD NN HD NG NI								
**Plus strand sequence**	T TAGGTGCTTTTCATTA gacatcaatgtcaga TAGCGACGCTGAAGA A								

Supplementary Figure S1. (A) The short genomic sequence of the *dsx1* locus used as the query for designing of TALEN. **(B)** Description of the chosen design, extracted from the output result of TALE-NT 2.0 web-based tool.

5’– (…)gccaagctcga T TAGGTGCTTTTCATTA gacatcaatgtcaga TAGCGACGCTGAAGA A tggtgagcaagggcgaggaggata
acatggccatcatcaaggagttcatgcgcttcaaggtgcacatggagggctccgtgaacggccacgagttcgagatcgagggcgagggcgagggccgcccctacgagggca
cccagaccgccaagctgaaggtgaccaagggtggcccccctgcccttcgcctgggacatcctgtcccctcagttcatgtacggctccaaggcctacgtgaagcaccccgccgac
atccccgactacttgaagctgtccttccccgagggcttcaagtgggagcgcgtgatgaacttcgaggacggcggcgtggtgaccgtgacccaggactcctccctgcaggacgg
cgagttcatctacaaggtgaagctgcgcggcaccaacttcccctccgacggccccgtaatgcagaagaagaccatgggctgggaggcctcctccgagcggatgtaccccgag
gacggcgccctgaagggcgagatcaagcagaggctgaagctgaaggacggcggccactacgacgctgaggtcaagaccacctacaaggccaagaagcccgtgcagctgc
ccggcgcctacaacgtcaacatcaagttggacatcacctcccacaacgaggactacaccatcgtggaacagtacgaacgcgccgagggccgccactccaccggcggcatgg
acgagctgtacaagtag ttgtttttgtttttttattattattattcattgatttattcttgctagcaagggatattattttagattatttgtttgtttgtttttaacatttgatgttgtttcatttttttttg
aatttatatcgtttctgcttctttatggcatcttttcttcgcactcttttattattgaaattagtgaaaaattccaatctgccaacgaaaaccccccttttgctttgattgatcttatagtaaccgaa
cgatttacatgtctgttgttcacacagcctgatggaaacatcctccaagtgattgttaagtgtaagatgattaccacctgagcttgtcacccgaaggaagaaaaacttgatgggtatatt
tttggaacttgattgatattgtgattttttttatctcattgaaattgaccgatt(…)gagtacgacttgtctccgatggtctaaccgtcttgttaaaagatgattaactgtaactcgaagagct
atggcttagtattttttttttctaatattcaattctacacttcattttccgataggcctatagatttaaaataaagtgtactatcaatgttagctactaatccatttaatggatcaattttggagttca
aaatctctcttgtttttaattacacgatgtttttggctgatgaaggaaaagttcaactgaaatgtatttctgaacatgcttaaaatatactgtgtctac ccggatcctgcacc(…) –3’

(= TALEN recognition site, = *mCherry* coding sequence, = *dsx1* 3’UTR)

Supplementary Figure S2. Description of donor vector’s sequence.

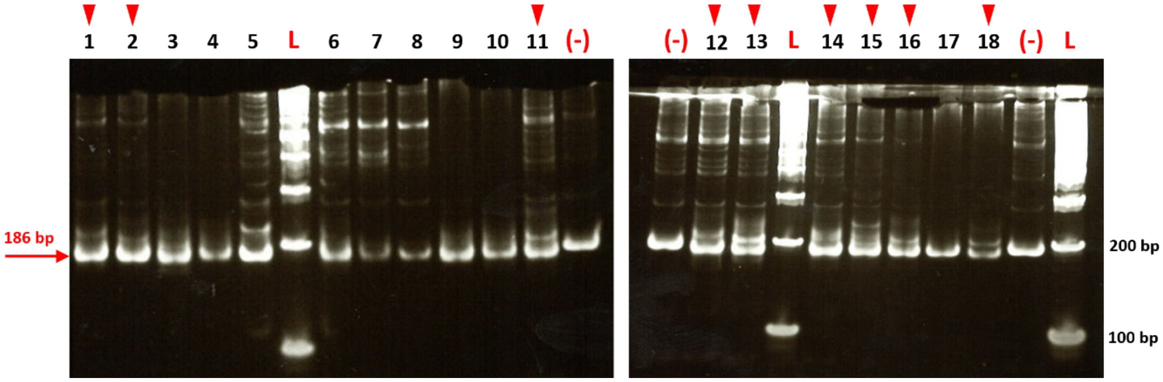

Supplementary Figure S3. *In vivo* functionality test of TALENs. Injection of TALEN mRNAs could cause a typical pattern of chimeric in-del mutation in the genome of 72 h juveniles, visible on a 20% polyacrylamide gel. **(-):** samples from non-injected embryos, showing the wild-type single band at 186 bp. **Red arrow heads:** cases with evident multi-banding pattern, a sign of in-del mutation. **L:** DNA ladder (the same type of ladder was used in both gels shown here, with the position of 100 bp and 200 bp bands indicated on the right edge of the figure). In this figure, among the 18 tested embryos, 9 (50%) showed clear signs of in-del mutation, indicating a fairly high efficiency of the TALEN design.

Injected solution	Injected eggs	72 h survivals	Adulthood survivals	Test 1 positive	Test 1+2 positive	Test 1+2+3 positive
**TALEN mRNAs** (500 ng/μL each) + **vector** (50 ng/μL)	83	31/83	18/31	3/18	2/18	**2**/18
		**37.3%**	**58.1%**	**16.7%**	**11.1%**	**11.1%**

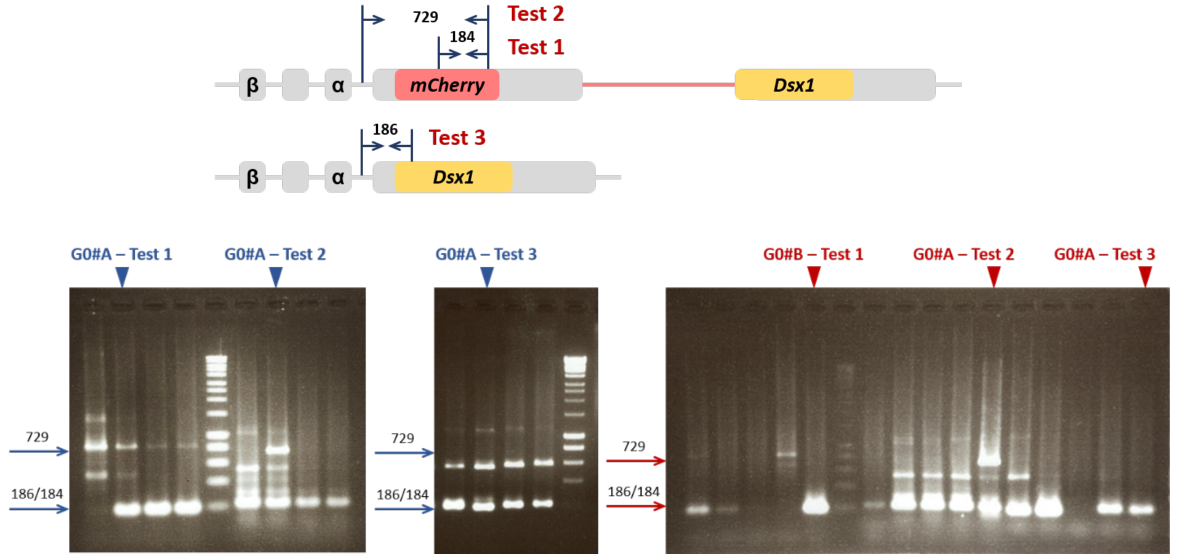

Supplementary Figure S4. Microinjection and screening result for G0 candidates. From 83 injected embryos, two survivors were found to be positive for all three tests described in the Methods section. Agarose gel electrophoresis results for each case are shown in this figure. The *dsx1* reporter strain, whose genotype is showed in **Figure 1B,** was established from **G0#B**.

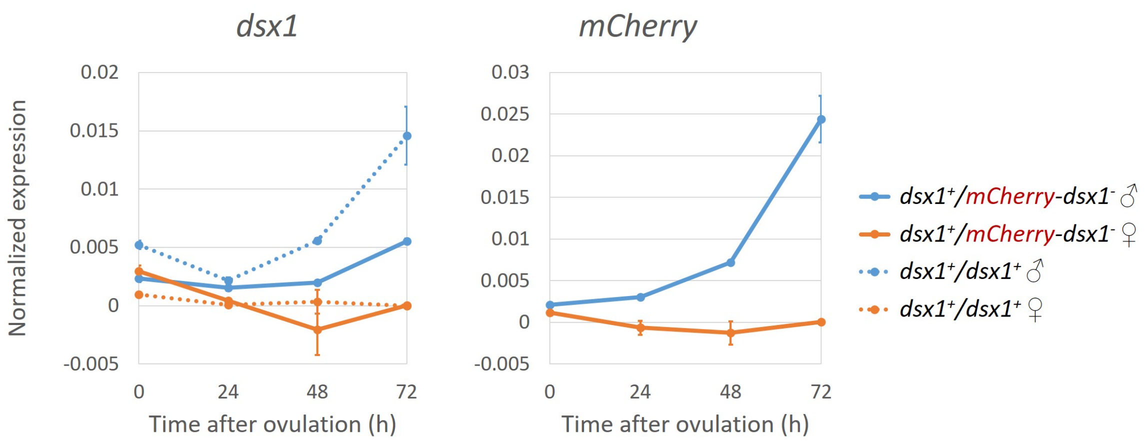

Supplementary Figure S5. Transcription of *dsx1* and *mCherry* in *dsx1*$^{+}$*/mCherry-dsx1*$^{-}$ reporter strain is consistent with wild-type pattern. Embryos of 0, 24, 48, and 72 hours post-ovulation were collected for the quantification of *dsx1* and *mCherry* expression levels. Primer pairs targeting the coding sequence of each gene were used. Normalisation was performed using *L32* (a ribosomal protein-encoding gene) expression as the internal control. N = 3. Error bars indicate standard error of the mean.

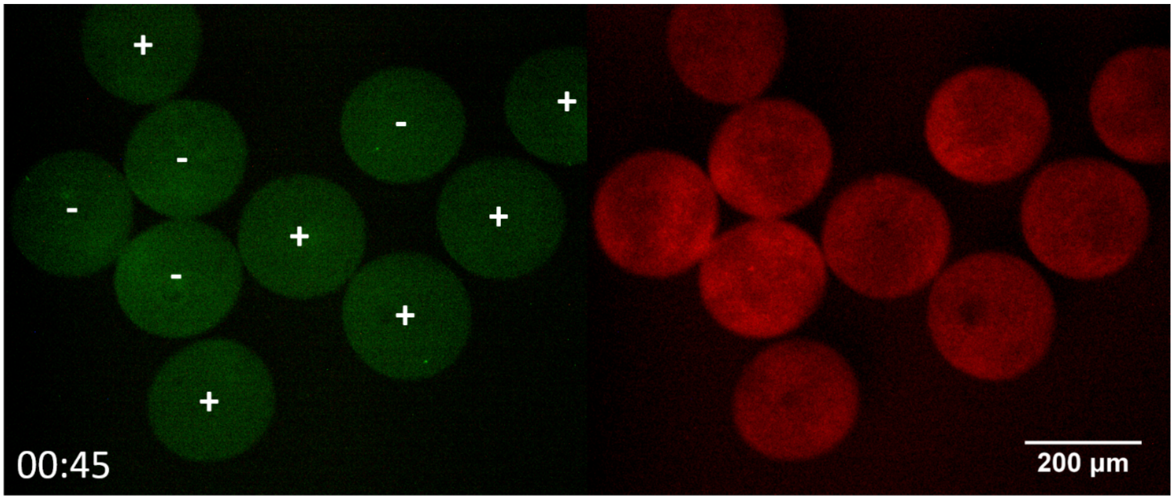

Supplementary Figure S6. An image from Movie S1. The image shown here is the first frame extracted from the movie, with extra labelling. **+**: males of *dsx1*$^{+}$*/mCherry-dsx1*$^{-}$. **-**: males of *dsx1*$^{+}$*/dsx1*$^{+}$ (both have *h2b-gfp* background). Lapsing clock in hh:mm format shows embryonic age (hours after ovulation).

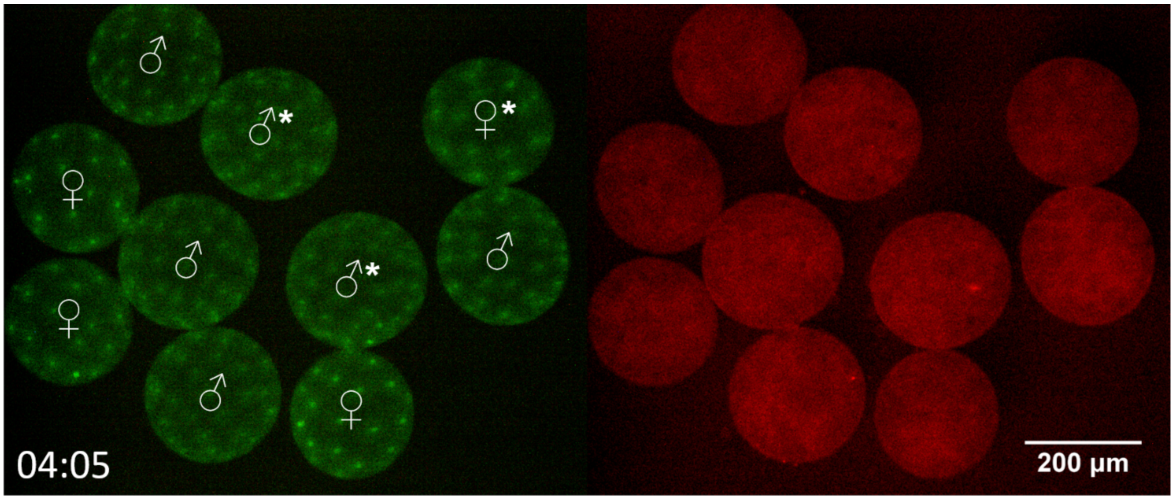

Supplementary Figure S7. An image from Movie S2. The image shown here is the first frame extracted from the movie, with extra labelling. ♂: males of *dsx1*$^{+}$*/mCherry-dsx1*$^{-}$. ♀: females of *dsx1*$^{+}$*/mCherry-dsx1*$^{-}$. *****: the three embryos shown in **Figure 8** and **Movie S3**. Lapsing clock in hh:mm format shows embryonic age (hours after ovulation).

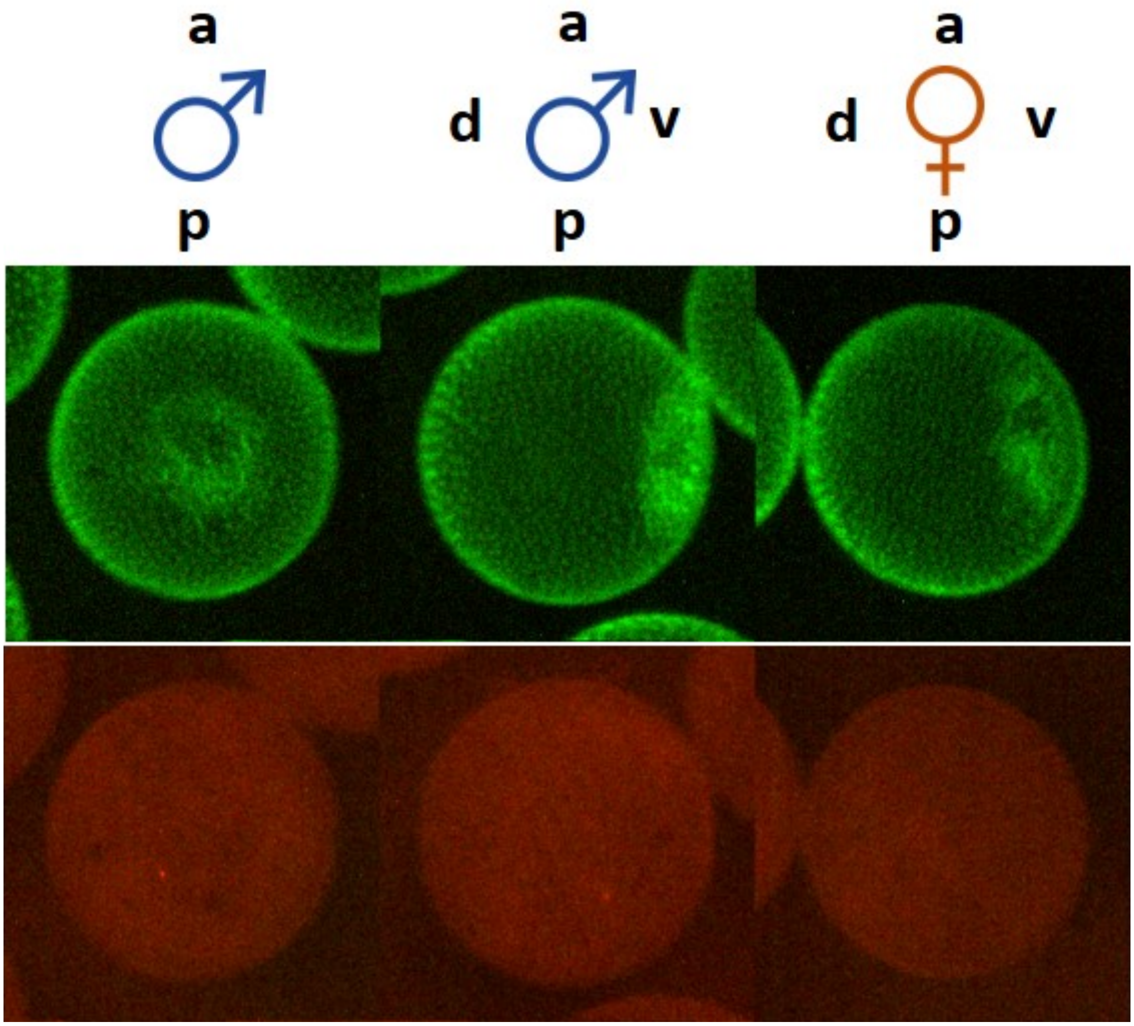

Supplementary Figure S8. A comparative close-in view of the three embryos indicated in Movie S2. See **Figure 8** and main text for description. **a:** anterior, **p:** posterior, **v:** ventral, **d:** dorsal.

Supplementary Movie S1. Reporter's activity during embryogenesis of *dsx1* reporter strain. Photos were taken every 7 minutes under GFP and mCherry filters. Ambient temperature was maintained at 22–23 °C. Please see Figure S6 and its caption for detailed information about this movie.

Supplementary Movie S2. Reporter's activity during embryogenesis of *dsx1* reporter strain. Photos were taken every 7 minutes under GFP and mCherry filters. Ambient temperature was maintained at 22–23 °C. Please see Figure S7 and its caption for detailed information about this movie.

Supplementary Movie S3. Appearance and movement of the first *dsx1* expressing cell cluster. The movie starts at gastrulation and ends at post-naupliar segmentation, showing a comparative close-in view of the three embryos indicated in **Movie S2**. See **Figure 8** and main text for description.
