# Supplementary figures and images for: Mapping the expression of the sex determining factor Doublesex1 in Daphnia magna using a knock-in reporter
Source: Sci Rep. 2017 Nov 2;7:13521. doi: 10.1038/s41598-017-13730-4 (PMC5668254; doi:10.1038/s41598-017-13730-4)

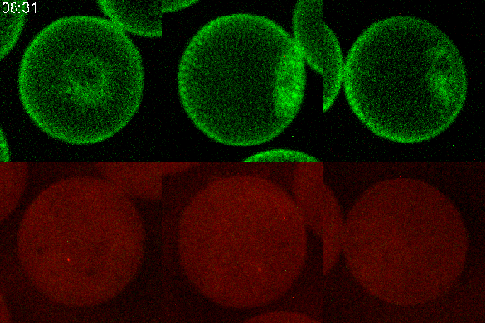

Supplement: Supplementary file 4 — Supplementary Movie S3 [file 41598_2017_13730_MOESM4_ESM.gif]
